# The Lifestyle Switch Protein Bd0108 of *Bdellovibrio bacteriovorus* Is an Intrinsically Disordered Protein

**DOI:** 10.1371/journal.pone.0115390

**Published:** 2014-12-16

**Authors:** Gerd Prehna, Benjamin E. Ramirez, Andrew L. Lovering

**Affiliations:** 1 Center for Structural Biology, Research Resources Center, University of Illinois at Chicago, Chicago, Illinois, United States of America; 2 Department of Microbiology and Immunology, University of Illinois at Chicago, Chicago, Illinois, United States of America; 3 Department of Biochemistry and Molecular Genetics, University of Illinois at Chicago, Chicago, Illinois, United States of America; 4 Institute of Microbiology & Infection, School of Biosciences, University of Birmingham, Birmingham, United Kingdom; University of South Florida College of Medicine, United States of America

## Abstract

*Bdellovibrio bacteriovorus* is a δ-proteobacterium that preys upon *Salmonella spp.*, *E. coli*, and other Gram-negative bacteria. *Bdellovibrio* can grow axenically (host-independent, HI, rare and mutation-driven) or subsist via a predatory lifecycle (host-dependent, HD, the usual case). Upon contact with prey, *B. bacteriovorus* enters the host periplasm from where it slowly drains the host cytosol of nutrients for its own replication. At the core of this mechanism is a retractile pilus, whose architecture is regulated by the protein Bd0108 and its interaction with the neighboring gene product Bd0109. Deletion of *bd0108* results in negligible pilus formation, whereas an internal deletion (the one that instigates host-independence) causes mis-regulation of pilus length. These mutations, along with a suite of naturally occurring *bd0108* mutant strains, act to control the entry to HI growth. To further study the molecular mechanism of predatory regulation, we focused on the apparent lifecycle switch protein Bd0108. Here we characterize the solution structure and dynamics of Bd0108 using nuclear magnetic resonance (NMR) spectroscopy complemented with additional biophysical methods. We then explore the interaction between Bd0108 and Bd0109 in detail utilizing isothermal titration calorimetry (ITC) and NMR spectroscopy. Together our results demonstrate that Bd0108 is an intrinsically disordered protein (IDP) and that the interaction with Bd0109 is of low affinity. Furthermore, we observe that Bd0108 retains an IDP nature while binding Bd0109. From our data we conclude that *Bdellovibrio bacteriovorus* utilizes an intrinsically disordered protein to regulate its pilus and control predation signaling.

## Introduction

*Bdellovibrio bacteriovorus* is a δ-proteobacterium that can either persist as a biofilm and filamentous cells in culture axenically, or as free swimming and swarming predatory bacteria [1], [2]. In the axenic state, *Bdellovibrio spp.* replicate readily without prey and as expected downregulate genes implicated in prey-location, while simultaneously upregulating genes involved in general growth and cell-division [1], [3]. This axenic growth is also referred to as the Host-Independent state (HI). In the predatory, or Host-Dependent (HD) state, *B. bacteriovorus* can no longer replicate autonomously and instead seek out and invade Gram-negative bacteria, including species known to be human pathogens [4]–[6]. Upon contact with a host bacterium, *B. bacteriovorus* attaches to the cell surface and penetrates the outer-membrane, burrowing into the host periplasm [7], [8]. Simultaneously, the cell wall hydrolases that mediate this process induce the host bacterium to round-up as the internalized predator attaches to the prey’s inner-membrane, forming the “bdelloplast” [9], [10]. Here, *B. bacteriovorus* effectively drains the host cytosol of nutrients, proteins, and nucleotides to fuel its own growth and replication in a controlled manner [10], [11]. After the host resources have been exhausted, the newly replicated progeny reach maturity as characterized by both septation and the secretion of multiple flagella, followed by the lysis of the host outer membrane [4], [12].

Similar to the HI state, the HD state and its various stages are characterized by the differential expression of a number of genes [1], [13]. Of particular interest are those genes and the subsequent encoded proteins that mediate the switch from the HI to HD lifestyle. Critical to this switch mechanism is a locus of genes that encode a Type IVb pilus and a distally located gene encoding a Type IVa pilin, that function in the attachment to Gram-negative bacteria and initiate the subsequent invasion [13], [14]. *Bdellovibrio bacteriovorus* that fail to properly secrete a pilus lack the ability to enter into prey [10] and thus do not progress into the HD lifestyle [8], [14], [15].

Pili structures are found throughout bacteria and archaea, and have diverse roles including motility, host cell recognition, and pathogenesis [16]. At the molecular level, Type IV pili generally consist of 8 or more genes [17], function in conjugation [18], cell-cell and surface adhesion [16], [19], and in the secretion of proteins [16], [17]. The gene products can be divided into the major and minor pilin proteins, where the major group consists of the higher abundant and core structural proteins of the apparatus, and the minor pilins at lower abundance that provide a regulatory function [16]. The Type IV pilus spans both the inner and outer membrane and is secreted into the extracellular environment under strict regulation, often dependent upon environmental cues such as temperature, pH, or the presence of a potential host [16], [17]. Most of the secretion components are localized to the bacterial inner membrane and periplasm, where they function in the assembly of the apparatus [17]. The extending pilus structure is formed from the secretion and oligomerization of the structural protein pilin [16]. Furthermore, work in *Pseudomonas aeruginosa* suggests that the minor-pilin proteins (such as FimU and PilX) interact with the extending pili and are used to regulate and control pilus length [20].

The *B. bacteriovorus* HD100 Type IVb pilus is predicted to consist of eight different genes (*bd0110-bd0114*, *bd0118, bd0119*, and *bd1290*) [13], and is regulated by two adjacently encoded proteins, Bd0108 and Bd0109 [14]. The major core subunit and structural component of the pilus is thought to be *bd1290*, encoding a PilA homolog [8], however both *bd0118* and *bd0119* also encode the major pilin homologues Flp1 and Flp2 respectively [13], [19]. Bd0110 and Bd0111 share similarity to TadA and TadB [13], which are ATPases that provide the energy for secretion, and Bd0113 and Bd0114 are thought to participate in the assembly process, with all four proteins localized to the predator’s inner membrane [14]. Additionally, the gene *bd0112* shares homology with *pilQ*, which functions to both anchor the pilin structure in the outer membrane and to allow passage through the membrane [13], [17]. It is important to note however, that HD100 also contains a putative Type IVa pilus consisting of the structural genes *bd1509–1512, bd0867 (pilQ), bd1585 (pilM), bd2167 (pill),* and *bd3852 (pilT)* [14].

**Figure 1:**
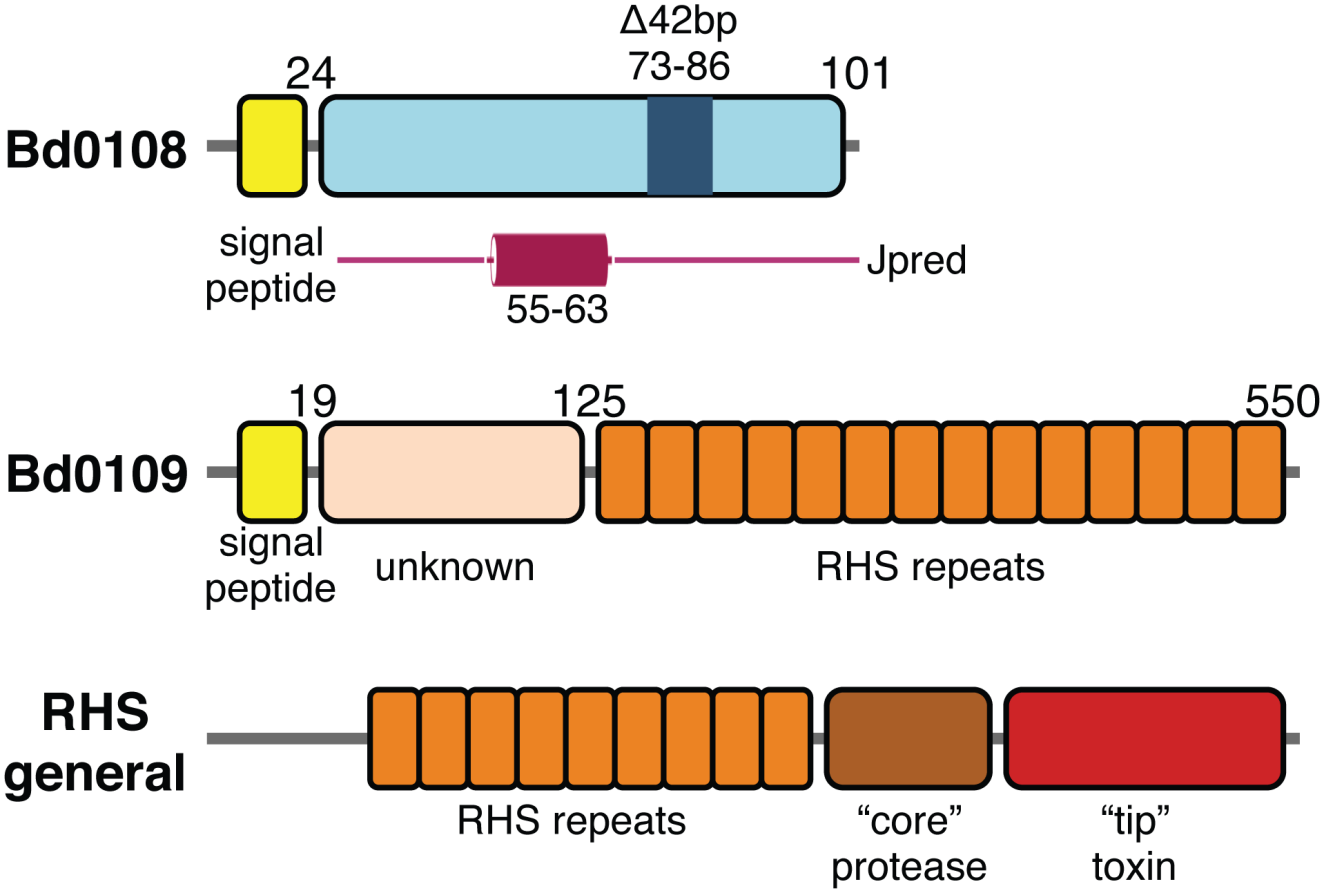
Domain map of Bd0108 and Bd0109. Schematic of Bd0108 and Bd0109 based on sequence homology and structure prediction with a comparison to RHS family proteins. Bd0108 is annotated nothing the region corresponding to the HI Δ42 bp deletion (above) and secondary structure prediction by Jpred (below). The magenta cylinder represents the predicted α-helix. Bd0109 is shown to consist of an N-terminal domain of unknown function and a C-terminal domain consisting of approximately 13 RHS repeat elements shown as orange boxes. The signal peptide for both Bd0108 and Bd0109 is displayed as a yellow box. RHS general represents a basic schematic for the domain organization of the RHS protein family.

The proteins Bd0108 and Bd0109 interact directly and work in concert to both promote the secretion of the *B. bacteriovorus* pilus and to regulate pilus length [14]. Bd0108 is a 101 amino acid protein with no predicted homology to known protein families [13], whereas Bd0109 is a 62.5 kDa protein that shows homology to recombination hot spot (RHS) domains [14] (Fig. **1**). RHS domains have many diverse roles such as regulation of pili [21], sugar binding [22], intercellular competition [23], and in secretion of ABC toxins [24]. For example, in another predatory bacteria *Myxococcus*, mutations in the putative RHS protein MXAN_6679 were attributed to the inability to retract pilus without methylcellulose stimulation [21]. In the case of the RHS proteins of an ABC toxin from *Yersinia entomophaga*, the B–C subunit proteins oligomerize into a hollow β-sheet shell which serves to encapsulate a protein partner for protection, folding and secretory purposes [24].

Although Bd0108 shows no homology to known protein sequences or predicted protein folds, secondary structure analysis predicts it to be partially α-helical in nature (Jpred [25], Phyre [26]) (Fig. **1**). However, despite the lack of structural knowledge, it is a central regulatory element of the HI to HD lifestyle decision of *B. bacteriovorus* [14], [27]. Approximately 89% of the currently known isolates of *B. bacteriovorus* that appear locked in the HI lifestyle contain a mutation in *bd0108* [27]. Furthermore, 46% of those strains demonstrate a specific mutation in *bd0108* consisting of an in-frame 42 bp deletion that removes residues 73–86 (*bd0108Δ42*) [27]. In fact, this deletion mutant was first characterized in the work of Cotter and Thomashow [15], where they named the *bd0108/bd0109* locus the host-interaction or *hit* locus. Moreover, subsequent genetic studies showed that the plasmid-based complementation of *bd0108* mutants reverts growth to the HD lifestyle and inhibits the growth of *B. bacteriovorus* in prey-independent environments [3], [14]. It is worth noting that in some HI strains (*e.g.* HID2, HID26 [1]), a wild-type *bd0108* sequence is present and other mechanisms enable the HD to HI conversion. These mechanisms may potentially center around RNA processing [3], and a small RNA has been shown to be transcribed from the hit locus [1]. Additionally, *Bdellovibrio* populations can be a mixture of HI and HD life-style participants. Dimorphic biofilms of *B. bacteriovorus* harbor a predatory population with wild type *bd0108* and a non-predatory population with both a wild type and a variable *bd0108* sequence [28].

At the molecular and cellular level, the expression of Bd0108 is directly related to the ability of *B. bacteriovorus* to extend and retract pili [14]. In wild-type HD predatory strains, *B. bacteriovorus* is observed to extrude pili with an average length of ∼0.47 µm. In contrast, *bd0108* deletion strains no longer secrete pili and in *bd0108Δ42* strains pili are observed but become misregulated, averaging ∼0.61 µm with 15% of observed pili being over 1 µm in length and much straighter [14]. Additionally, if HD cells are transformed with a plasmid bearing *bd0108* and its promoter region, not only is the frequency of observing pili increased 3-fold, but also the pili are longer than those for wild-type [14].

The current model for the regulation of the *B. bacteriovorus* pilus and the HI to HD lifestyle switch centers on the hypothesis that Bd0108 and Bd0109 function as a regulatory complex in the periplasm [14]. Both proteins contain an N-terminal secretion signal for cleavage by the Sec system [29] and secretion into the periplasm where they are thought to function and interact [14]. Additionally, Bd0109 is speculated to also anchor itself at the cell wall and simultaneously interact with the oligomerizing pilin proteins, perhaps to influence length. To regulate these processes, Bd0108 is thought to sequester Bd0109 or inhibit interaction with the pilus. This interplay together with the other protein components initiates a regulatory signal dependent upon extrusion and retraction of the pilus to mediate the switch into predatory intracellular growth [14]. In *bd0108* deletion strains Bd0109 is potentially unregulated, resulting in incorrect pilus mediated signaling for switching to the HD life-style. Likewise, in the *bd0108Δ42* strain, Bd0108 might still interact with Bd0109 to allow the formation of the pilus, but incorrect extrusion and retraction of the pilus signals *B. bacteriovorus* to maintain HI dependent growth [14].

Although several genetic studies have demonstrated that Bd0108 acts as a switch for the HI and HD lifestyle decision process of *B. bacteriovorus*, information is lacking about the biochemistry and structure of Bd0108. In this study, we undertake a structural and biophysical analysis of Bd0108. Here we use nuclear magnetic resonance (NMR) spectroscopy complemented by additional biophysical methods to probe the structure and dynamics of Bd0108. Together our results show that Bd0108 is an intrinsically disordered protein (IDP) with no regular secondary structure that persists as an extended conformational ensemble. Additionally, we probe the interaction between Bd0108 and Bd0109 in more detail using both isothermal titration calorimetry (ITC) and NMR spectroscopy. From our data we conclude that *Bdellovibrio bacteriovorus* utilizes an intrinsically disordered protein as a switching mechanism between the Host-Independent and Host-Dependent lifestyle. Furthermore, we hypothesize that Bd0109 encapsulates Bd0108 as part of the regulatory cascade controlling predation signaling.

## Results

### Purification and biophysical characterization of Bd0108

**Figure 2:**
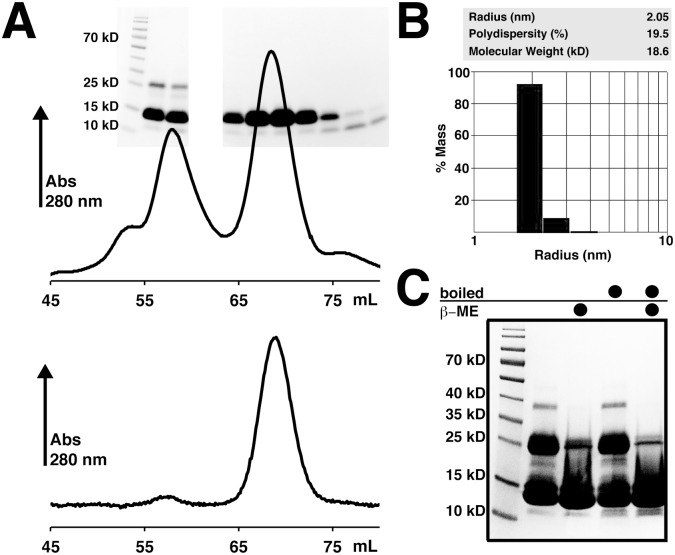
Purification of Bd0108. (A) Gel filtration profile of purified Bd0108. (Upper panel) Bd0108 elution from an SD75 column after affinity chromatography and cleavage of His-tag. Overlaid on the chromatogram is an SDS-PAGE gel showing purity of the corresponding fractions. (Lower Panel) Elution profile of the late elution peak re-run on an SD75 column. (B) Dynamic Light scattering of the Bd0108 late elution peak at approximately 2 mg/mL. (C) SDS-PAGE gel of purified Bd0108 with and without reducing agent (β-mercaptoethanol) and/or boiling indicated by a black circle.

Bd0108 can be readily purified to homogeneity, however as shown in Fig. **2A** initial gel-filtration results showed the presence of two major Bd0108 species of differing molecular weight. Separation over a 16/60 SD75 Superdex column yielded predicted molecular weights (MW) of approximately 45 kDa and 23 kDa, corresponding roughly to a theoretical tetramer and dimer respectively. However, if the later eluting species of 23 kDa is isolated, concentrated, and eluted again from the size-exclusion column it remains a single species (Fig. **2A**). Additionally, dynamic light scattering (DLS) measurements of the small molecular weight species showed a non-aggregating homogenous species of Bd0108 in solution (Fig. **2B**). Furthermore, the DLS data closely match the size predicted from size-exclusion chromatography, yielding a molecular weight of 16.5 kDa (hydration 18.6 kDa) with low polydispersity, indicating that the small species is mono-disperse and is likely not in equilibrium with the larger species.

**Figure 3:**
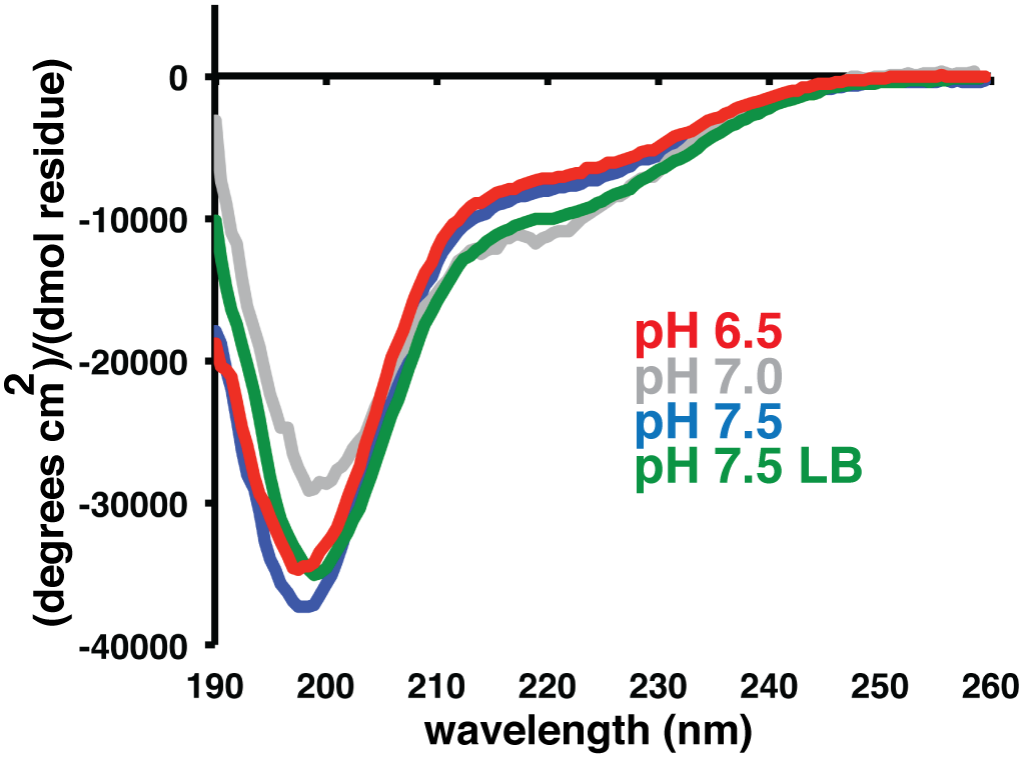
Bd0108 adopts an extended conformation. Circular Dichroism spectra of isotopically labeled Bd0108 recorded at pH 6.5 (red), pH 7.0 (grey), and pH 7.5 (blue). CD spectrum recorded at pH 7.5 of Bd0108 purified from *E. coli* grown in LB media (green).

Given that Bd0108 has a predicted disulfide by Metaldetector [30] (**S1** Figure), we hypothesized that the larger species may be a result of improper disulfide formation during over-expression and/or concentration. To test this, we ran an SDS-PAGE gel of Bd0108 treated with and without an excess of reducing agent β-ME (β-mercaptoethanol) to observe the presence of different covalently linked Bd0108 species (Fig. **2C**). As can be clearly observed in the lanes without β-ME, Bd0108 forms a ladder of at least three species (12 kDa, 24 kDa, and 36 kDa), in agreement with the gel-filtration profile. However, in the presence of reducing agent, the amount of higher molecular weight species is significantly lowered, showing that they are in fact dependent upon disulfide formation. Additionally, circular dichroism (CD) data show that Bd0108 adopts an extended conformation that is readily identifiable from the characteristic curve shape with a minimum near 200 nm (Fig. **3**). The DLS and CD data taken together demonstrate that Bd0108 is a monomer in solution (the MW predictions from the various techniques in Fig. **2** assume a globular fold and thus over-estimate weight).

### Bd0108 is an intrinsically disordered protein

**Figure 4:**
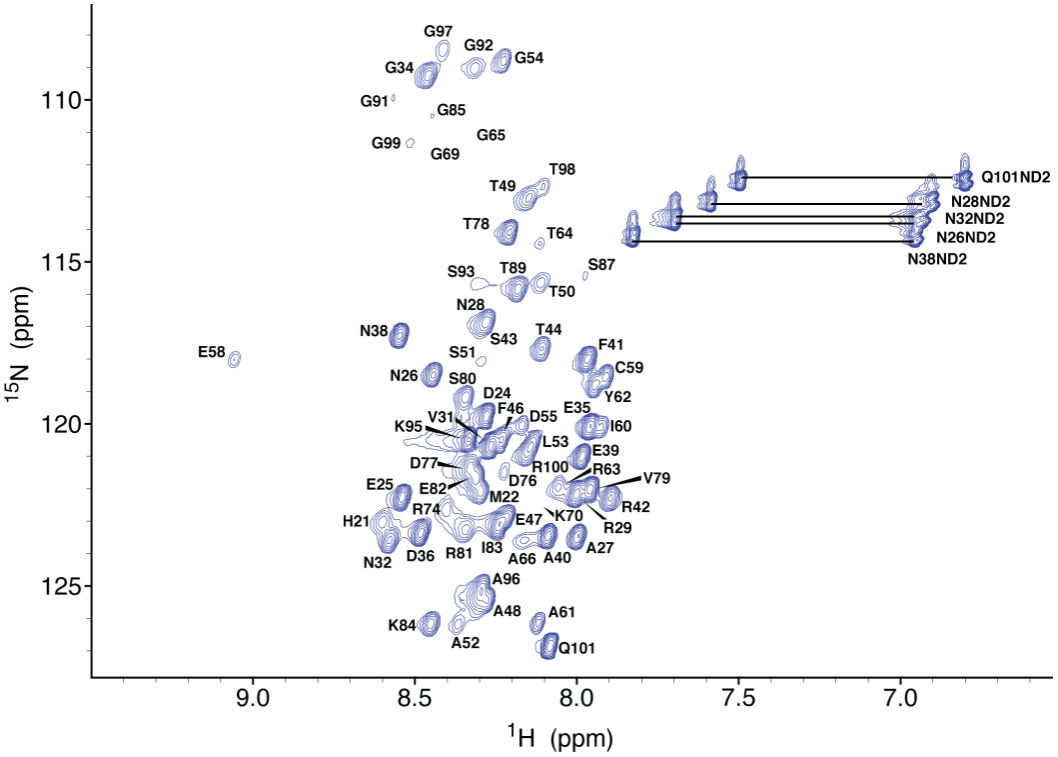
Bd0108 ^1^H-^15^N HSQC. Assigned ^1^H-^15^N HSQC of Bd0108 residues 23–101 taken at pH 7.0 and 25°C. The full chemical shift list can be accessed via the BMRB with accession code 25327.

**Figure 5:**
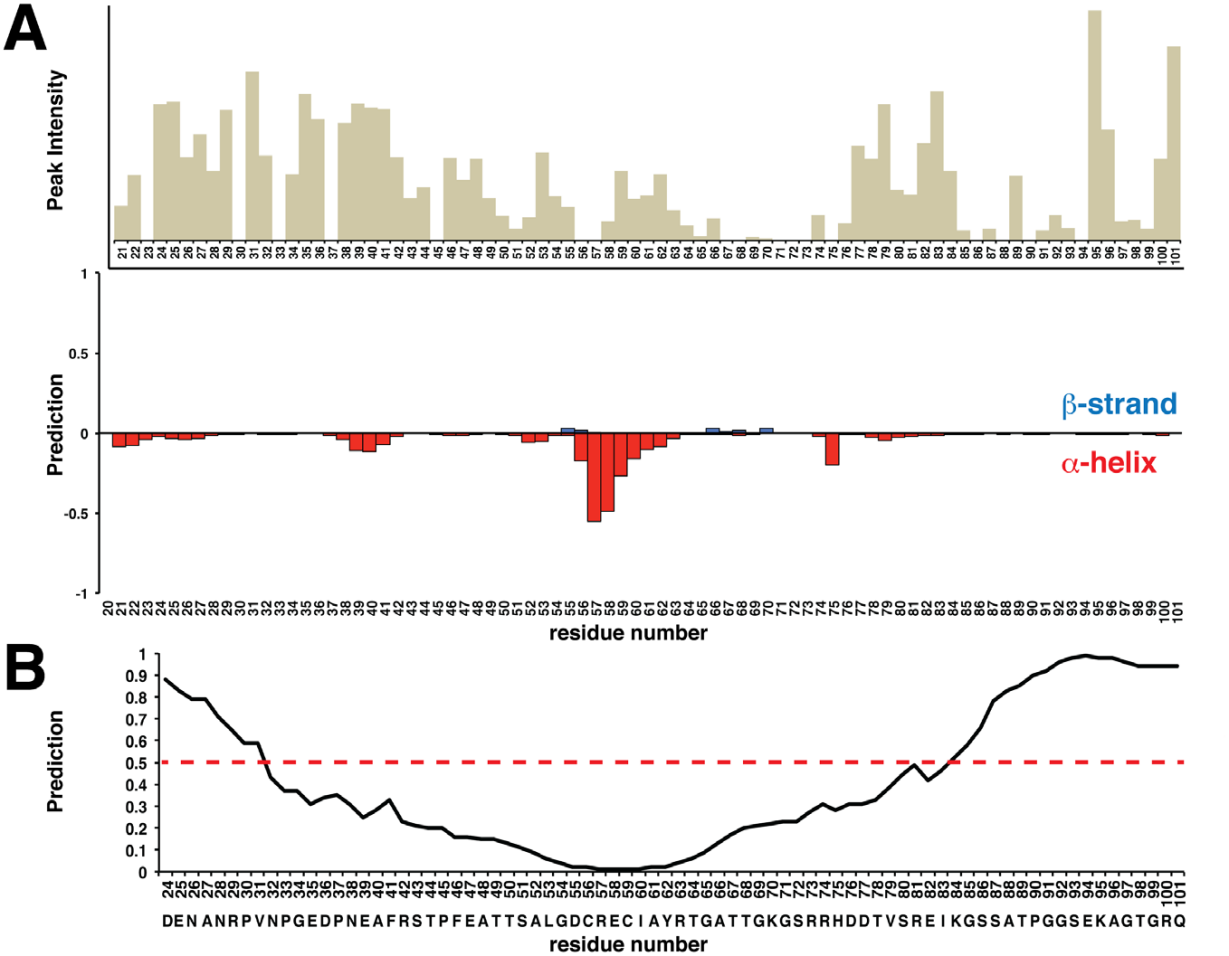
Bd0108 is an intrinsically disordered protein. (A) Top Panel: Relative peak height of observed backbone resonances recorded from a ^1^H-^15^N HSQC at pH 7.0 for each assigned residue. Bottom Panel: Artificial Neural Network (ANN) secondary structure prediction from assigned chemical shifts (Cα, Cβ, CO, H-N) calculated by Talos+. Values that are negative and red have α–helical character and values that are positive and blue have β–strand character. (B) Disorder prediction of Bd0108 from Disopred2. Higher values indicate greater likelihood of being disordered. The dotted line represents the 50% confidence level.

As initial crystallization trials were unsuccessful, we employed nuclear magnetic resonance (NMR) spectroscopy for structural studies of Bd0108. Bd0108 is extremely amenable to solution studies as it was stable for months, showing only a slight but stable degradation after a week (**S2A** Figure). Fig. **4** shows the assigned ^1^H-^15^N HSQC spectrum of Bd0108 (full chemical shift data available at the Biological Magnetic Resonance Bank (BMRB) accession code 25327). As can be noted from the spectrum, several expected H-N resonances are not visible and could not be assigned. Most notably this includes residues bordering the central predicted helical region, including residues 71–73 and 75 that overlap with the region of Bd0108 associated with the switch between the HI and the HD lifestyles (*bd0108Δ42 b*p or residues 73–86 and point mutants 70 and 72) [27]. A ^1^H-^15^N HSQC of Bd0108 recorded at pH 6 resulted in an enhancement of the signal for several glycine residues, but only a net gain of two strong peaks out of the nine unassigned residues (**S2A** Figure). This suggests that amide hydrogen exchange with solvent is not the only cause of the unobserved resonances. An experiment recorded at 40°C also did not result in additional peaks (**S2B** Figure), however at higher temperatures Bd0108 becomes unstable and begins to precipitate. Together, the data indicate that hydrogen exchange and likely conformational exchange occur in those regions of the protein [31], [32]. Further examination of the assigned chemical shifts show that the observed Cβ resonance for C59 was recorded at 37.5 ppm indicating that the thiol group is oxidized [33]. Although no chemical shift information is available for C56 as it is in a region of exchange (Fig. **4** and Fig. **5**), given the predicted disulfide (**S1** Figure) and the oxidation state of C59 Cβ, we conclude that Bd0108 does contain a disulfide between residues C56 and C59.

To further probe the structure of Bd0108, we calculated the secondary structure of Bd0108 from the chemical shift assignments using Talos+ [34] (Fig. **5A**) and also collected a ^15^N NOESY-HSQC experiment [35]. As shown in Fig. **5A**, the experimental chemical shift data does not predict any regular secondary structure for Bd0108. The overall character of Bd0108 does seem to tend towards α-helix, with residues 56–62 showing the greatest helical character, however the prediction from Talos+ is that of a dynamic backbone. Furthermore, the ^15^N NOESY-HSQC experiment yielded only one observable inter-residue NOE between E58 and C59 agreeing with the prediction from Talos+ that Bd0108 does not favor a single conformation. Regardless, even without dihedral angle restraints and NOE distance restraints, the chemical shift data was submitted to CS-Rosetta to attempt a structure calculation [36], [37]. In line with the experimental data, CS-Rosetta failed to calculate an ordered structure or even discrete clusters of similar energy structures (**S3** Figure).

For verification and support of the NMR and computation data, circular dichroism (CD) spectra of isotopically labeled Bd0108 were measured at pH 6.5, pH 7, and pH 7.5 (Fig. **3**). As indicated by the CD spectra, Bd0108 adopts an extended and disordered conformation at and near physiological pH. Furthermore, deconvolution of the spectra using Dichroweb [38] yields a ∼0.1 partition of α-helix and a ∼0.9 partition of ‘unordered’ in agreement with the chemical shift data (Fig. **5A**). Additionally, we measured the CD spectrum of Bd0108 produced from LB media instead of minimal media to control for mis-folding due to a possible lack of enough trace metals and/or the additional stress placed upon *E. coli* grown in these conditions. As shown in Fig. **3**, the spectrum is identical to that collected from the isotopically labeled sample. Moreover, as the natural environment of Bd0108 is the periplasm [14], we also collected a ^1^H-^15^N HSQC of Bd0108 purified from a vector with an N-terminal PelB tag for direct targeting during expression to the periplasm [39]. Again, the observed spectrum is identical to the cytosolic purification (**S2C** Figure), suggesting that the disordered nature of Bd0108 is not due to lack of folding by a specific chaperone or disulfide isomerase located in the periplasm [40], at least in this recombinant system.

The sum of the biophysical data collected (Figs. **2** **–** **5**) show that Bd0108 is an intrinsically disordered protein (IDP) that adopts an extended conformation without discrete secondary structure. It is of importance to note however, that Bd0108 is predicted to have a central ordered region of 20–30 amino acids according to Disopred2 [41] (Fig. **5B**) and has a complex, non-repetitive sequence uncharacteristic of many IDPs [42].

### Dynamic Properties of the Bd0108 backbone

**Figure 6:**
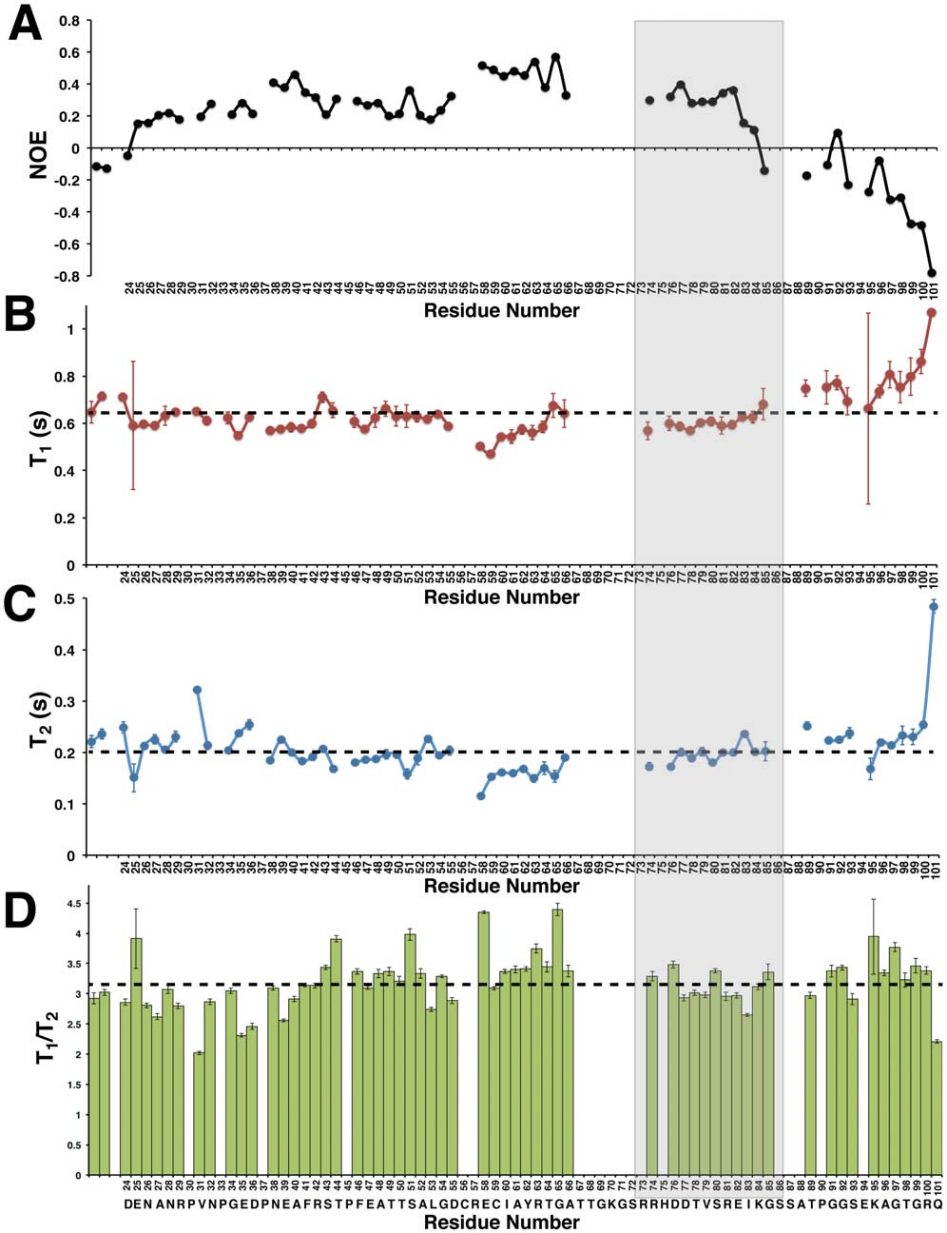
Bd0108 backbone dynamics. (A) ^1^H-^15^N heteronuclear NOE relaxation data (B) T_1_ data (C) T_2_ data (D) Ratio of T_1_ to T_2_. All experiments were taken at 25°C at pH 7.0 with an 800 MHz Bruker Spectrometer. Values and errors were calculated using Sparky and are plotted by residue number. The dashed lines represent the average T_1_ time (640 ms), T_2_ time (203 ms), and T_1_/T_2_ ratio mean. The region corresponding to the *bd0108Δ42 bp* deletion mutant that lacks residues 73–86 is outlined in grey.

As we have shown that Bd0108 is an IDP, we acquired a set of NMR relaxation experiments to probe the backbone dynamics of the protein. We collected ^1^H-^15^N NOE, longitudinal or spin-lattice (T_1_), and transverse or spin-spin (T_2_) relaxation data of the Bd0108 monomer (Fig. **6**). These three parameters are measured per residue (H-N resonance) and typically considered together to gauge the flexibility of a protein backbone and regions of local order and disorder [43], [44]. The average values observed at 25°C and 800 MHz field strength for Bd0108 are 0.31 (positive NOE), a T_1_ of 640 ms and a T_2_ of 203 ms. The ^1^H-^15^N NOE relaxation experiment in particular is useful for determining regions of a protein that exhibit nanosecond-picosecond dynamics (negative NOE peak) and those residues that adopt a stable conformation (NOE>0.6) [45]. As shown for Bd0108, no region of the protein appeared as a stable conformation. Overall the protein displays small but positive NOEs indicative of areas of transient structure, with the C-terminus (residues 85–101) showing negative NOEs representing ns-ps time scale movements. Residues 58–66 showed the highest NOE values (∼0.5) and were flanked by regions of hydrogen and intermediate exchange [32] (**S2A** Figure), suggesting that they spend a significant fraction of their time sampling a stable conformation (Fig. **6A**). Considering the chemical shift data and the secondary structure calculation by Talos+ (Fig. **5A**), residues 56–66 likely sample an α-helix conformation. This is supported by the CD data (Fig. **3**) that allows for an approximate 10% partition of α-helix. Furthermore, these observations are in agreement with the relative peak intensity of each residue (Fig. **5A**). The residues with the highest intensity correspond to regions of ns-ps timescale movements and disorder, compared to the central region of Bd0108 that has a lower average intensity and is predicted to be able to adopt an ordered conformation (Fig. **5** and Fig. **6A**).

When considering both the T_1_ and T_2_ data (Fig. **6B** and Fig. **6C**), there seems to be no area of significant variation from the mean values, except slightly for residues 58–66 and the C-terminus. Lower than mean T_1_ and T_2_ values are observed for the residues 58–66 suggesting some local structure, and as expected high T_1_ values indicative of fast time-scale movements for the C-terminal region. This agrees with both the ^1^H-^15^N NOE relaxation data (Fig. **6A**) and the secondary structure and order predictions (Fig. **5**). A plot of the T_1_/T_2_ ratios per residue potentially show a slight increase for the central residues relative to the average, but overall there are no regions of significant difference from the mean (Fig. **6D**). This suggests that as a whole, the backbone residues of Bd0108 have roughly the same degree of high flexibility [43].

### Biochemical Characterization of the Interaction between Bd0108 and Bd0109

**Figure 7:**
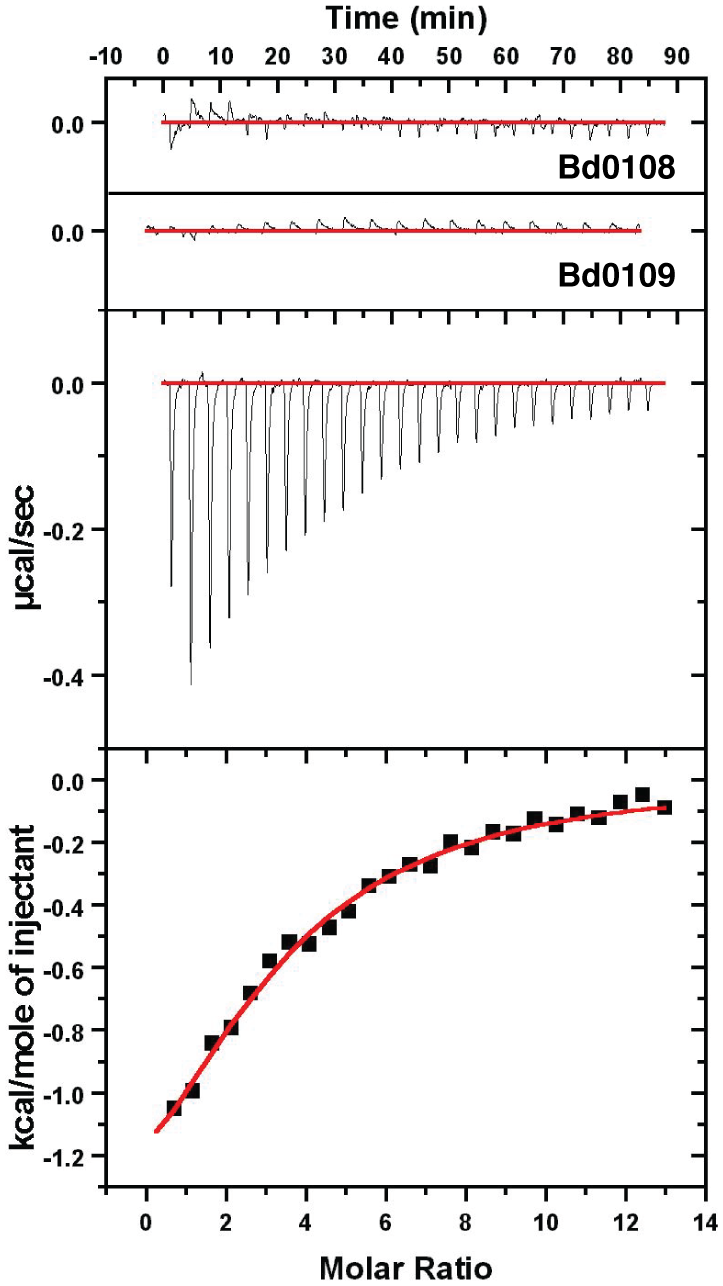
Isothermal titration calorimetry of Bd0108 and Bd0109. The top two panels show heats from control titrations of Bd0108 into experimental buffer and experimental buffer into Bd0109. The middle panel shows heats of titration of 1.2 mM Bd0108 into 20 µM Bd0109, and the bottom panel calculated enthalpies after data correction in origin software. The resulting stoichiometry (N-value) is 3.1, K_d_ of 45 µM, ΔH of −2075 kcal/mole, ΔS of 12.92 cal K^−1^ mol^−1^.

**Figure 8:**
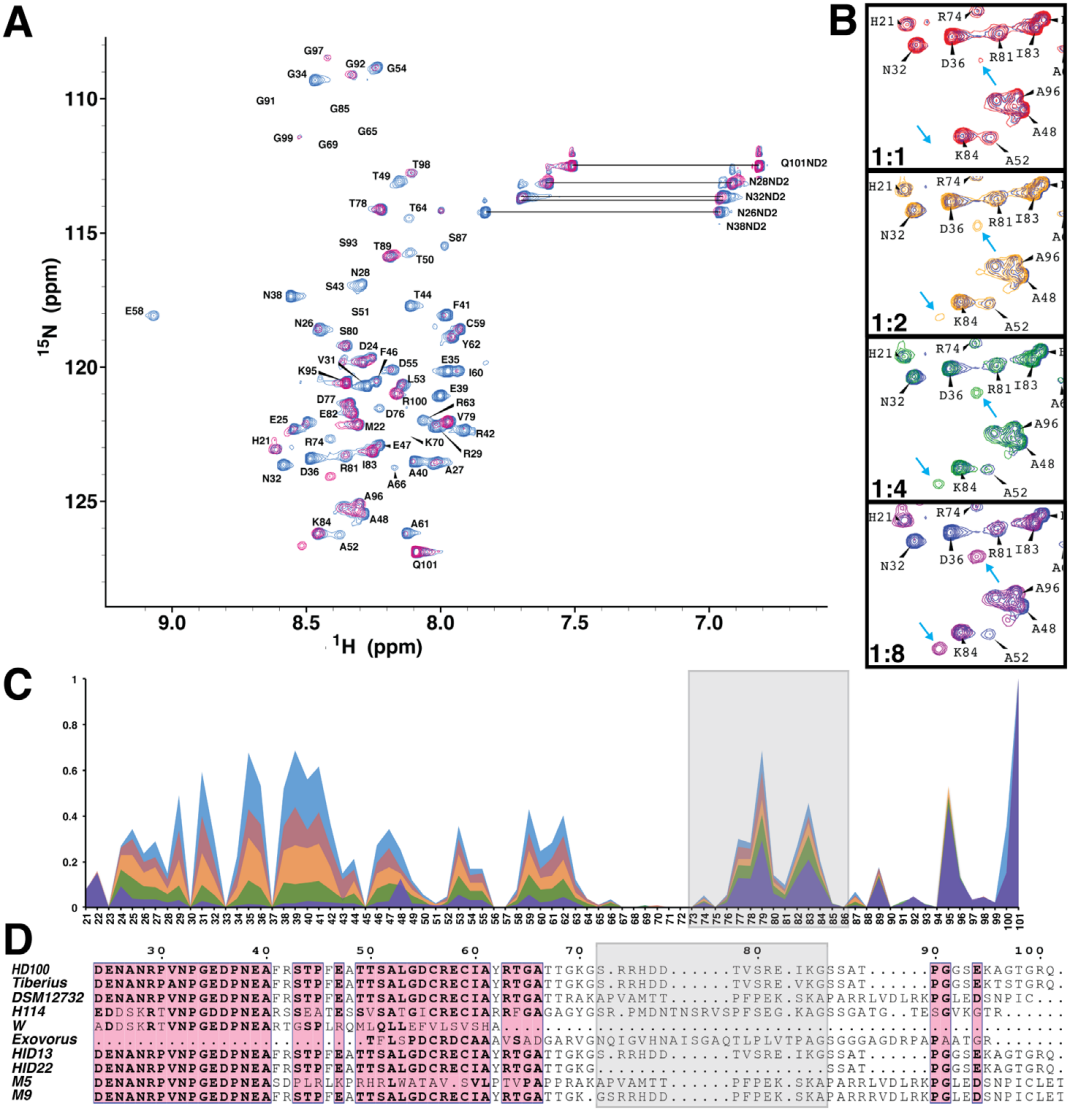
NMR titration data of Bd0108 and Bd0109. (A) ^1^H-^15^N HSQC of 25 µM Bd0108 titrated with 200 µM Bd0109. Blue is Bd0108 with 0 µM Bd0109 and magenta is with 200 µM Bd0109. (B) Close-up of region ^1^H-^15^N HSQC of 25 µM Bd0108 titrated with increasing amounts of Bd0109 at the ratio shown in each panel. Arrows indicate the dose dependent appearance of new peaks in the spectra. (C) Graph representation of peak data height for each residue in the presence of 0 µM Bd0109 (blue), 25 µM Bd0109 (red), 50 µM Bd0109 (orange), 100 µM Bd0109 (green) and 200 µM Bd0109 (purple). Peak height has been normalized to the peak height of the C-terminal residue Q101 for each spectrum. (D) Sequence alignment of Bd0108 from known strains mapping sequence conservation. Strain HID22 is the *bd0108Δ42 bp* deletion mutant that lacks residues 73–86. The region corresponding to residues 73–86 is outlined in grey in both panels C and D. The alignment was created using Clustal Omega (http://www.ebi.ac.uk/Tools/msa/clustalo/) and Espript 3.0 (http://espript.ibcp.fr/ESPript/ESPript/).

With the structure of Bd0108 characterized and the backbone chemical shift assignments known, we wanted to explore in detail the interaction with its known binding partner Bd0109. Although previous work had used both fluorescence quenching and protease protection assays between purified Bd0108 and Bd0109 to demonstrate an interaction [14], the mechanism remains unknown. To further expand upon the previous results, we examined the interaction of Bd0108 to Bd0109 by both isothermal titration calorimetry (ITC) (Fig. **7**) and NMR titration experiments (Fig. **8**).

For ITC experiments, the titration of concentrated Bd0108 into purified Bd0109 proceeded to saturation, with the control titrations of both Bd0108 and Bd0109 showing no significant background heats (Fig. **7**). The resulting curve yields a dissociation constant of 45 µM with an observed stoichiometry of ∼3∶1 (Bd0108:Bd0109). A high micro-molar K_d_ can be characteristic of transient interactions, such as those involved in signaling or secretion pathways [46], and also low affinity yet specific binding mechanisms [47]. These modes of protein binding often employ protein disorder and dynamics [48]. The observed enthalpy of the reaction was negative (−2075 kcal/mole) whereas the entropy was calculated to be positive (12.92 cal K^−1^ mol^−1^), showing that both hydrogen bonding and hydrophobic interactions drive the reaction. One might have expected the resultant entropy to be negative (unfavorable), as Bd0108 would be expected to lose conformational freedom, however this can be explained from compensation due to the hydrophobic effect from the aliphatic and aromatic residues of Bd0108 de-solvating upon binding Bd0109.

In an attempt to map those residues of Bd0108 that interact with Bd0109, we titrated increasing amounts of Bd0109 into ^15^N labeled Bd0108 and recorded the subsequent ^1^H-^15^N HSQC (Fig. **8**). As shown in Fig. **8A** and in Fig. **8C** the signal from the N-terminal residues 24 to 66 decreases drastically, losing 90% to 100% of the observed signal in a dose dependent manner. The effect is especially striking upon visual inspection of the ^1^H-^15^N HSQC, where the N-terminal resonances appear to vanish from the spectrum (Fig. **8A**). In contrast, the C-terminal residues 74–85 showed only moderate signal loss with residues 89–101 being completely unaffected (Fig. **8C**). Moreover, the assigned cloning artifacts (residues 21 and 22) also showed no loss of signal throughout the titration experiment. These observations demonstrate that the N-terminal residues of Bd0108 enter into chemical exchange in the presence of Bd0109, likely on a µsec to msec time scale [32], consistent with the low affinity interaction observed in the ITC binding data (Fig. **7**). Furthermore, the data also suggests that the N-terminal residues 24–66 are the site of binding to Bd0109 and/or adopt new conformation(s) as part of the interaction mechanism. These observations are further underscored and supported by the fact that for the currently sequenced strains of *B. bacteriovorus*, the conserved sequence regions map to the N-terminus of Bd0108, particularly residues 32–40 (Fig. **8D**).

Although the most significant effect observed in the NMR titration experiments was the loss of signal largely due to intermediate chemical exchange, two new resonances did appear in a dose dependent manner. As shown in Fig. **8B**, near the assigned resonances for D36 R81 and K84, two strong peaks become observable. The obvious candidates for these new signals are N32, D36, and A52 as they are proximal and disappear at the same rate the new peaks are forming. This is an especially tempting assignment for both N32 and D36, which are conserved in *B. bacteriovorus.* However, given that several residues could not be assigned and are in conformational exchange before titration with Bd0109 (Fig. **4** and Fig. **6**), we cannot rule out that these new resonances are not instead from those residues.

## Discussion

In an attempt to further understand the mechanism of the HD to HI lifestyle switch of *Bdellovibrio bacteriovorus* at the molecular level, we have structurally characterized the protein Bd0108 using a wide variety of biophysical techniques. We demonstrate that Bd0108 is a monomer in solution by using both size-exclusion chromatography and dynamic light scattering. This conclusion is further supported by our biochemical studies showing that the monomer of Bd0108 is active as it binds to Bd0109 as measured by both isothermal titration calorimetry studies and by NMR titration experiments. These interaction studies have also granted insight into the molecular details of the potential *B. bacteriovorus* pilus regulatory complex formed between Bd0108 and Bd0109, and thus predation as a whole. Moreover, through the use of NMR spectroscopy we probe the structure and solution state dynamics of Bd0108, revealing that Bd0108 is an intrinsically disordered protein.

Intrinsically disordered proteins (IDP) and intrinsically disordered regions of proteins (IDPR) are omnipresent throughout biology and often function in regulation and signaling reactions [48]–[50]. IDPs and IDPRs exist in a high-energy state as an ensemble of a few discrete conformations, many low populated states, or near exclusively as random coil [49], [51]. Interaction with a ligand or a binding partner can induce a transition to an ordered conformation, as observed for eukaryotic initiation factor [52] and the KIX domain that folds into two α-helices upon binding the transcription factor CREB [53]. Alternatively IDPs and IDPRs may never adopt a stable low-energy folded state, and instead provide a transient interaction to allow multispecificity with one or more binding partners to facilitate a biological response or reaction [48]. Examples of this mode of action include the secreted bacterial protein YebF [31] and the inhibitor of cyclin dependent kinase, Sic1 [54]. Additionally, the flexibility provided by IDPRs can be used for conformational freedom for domain movements as observed in the *Salmonella* virulence regulator protein ZirS [45] and the E3 ubiquitin ligase Smurf2 [55]. Despite these many examples, the field of IDP structural biology is relatively new, with the detailed study of protein disorder and its intimate relationship to biological function only recently appreciated [48].

The summation of both our NMR and CD data show that Bd0108 is an IDP that in solution exists almost exclusively as random coil. This is not only evident from the chemical shift data (Fig. **5A**) and the characteristic random-coil CD spectra (Fig. **3**), but also from the obtained NMR relaxation data (Fig. **6**). However, the biophysical data do suggest that Bd0108 may show some conformational specificity in the central region of the molecule at residues 56–66. Specifically, both the chemical shift data and the deconvolution of the CD spectra indicate a small amount of helical conformation. Furthermore, in this region the Cβ shift of C59 indicates that the thiol group is oxidized and forming a disulfide with C56. As these residues are separated by four positions (CXXC motif), their side-chains would be placed adjacent on the same face of an α-helix. This suggests that the function of the Bd0108 disulfide may be an anchor or at least be the initial seed to tip the conformational equilibrium to that of an α-helical conformation, perhaps in preparation of folding when encountering a currently unknown binding partner. However, the relaxation data clearly show a highly flexible backbone, with no low-energy long-lived stable conformation (Fig. **6**). From this analysis we conclude that Bd0108 exists as a conformational ensemble of mostly random coil, with a slight propensity for α-helix at its core.

A survey of the Protein Data Bank using PDBeMotif [56] to find structures containing a CXXC disulfide-linked motif in an alpha helical environment returns several hits, including thioredoxin-like proteins (*e.g.* NrdH, accession code 4K8 M [57]), ALR proteins (3MBG [58]) and MerP metal ion-binding domains (1OSD [59]). These three all have different folds, but place the CXXC motif at the start of the alpha helix, and use the cysteine residues as a redox-sensitive signaling motif. The use of IDPs and IDPRs is common in redox based signaling pathways [60], highlighting the possibility that Bd0108 may provide such a function. Other non-redox active folds do also possess CXXC-initiated helices, but the enrichment in sensory functions of proteins containing this motif may hint at such a role for Bd0108. However, the homologue in *Bdellovibrio* strain W is lacking conservation in this region (Fig. **8D**).

Central to predation in *B. bacteriovorus* is the proper extrusion and retraction of a Type IVb pilus, which mediates the signal to switch between the HI and HD lifestyle. At the genetic level it was shown that *bd0108* is critical to this process, with its deletion and naturally isolated mutations inhibiting predation [27]. Our interaction studies have further delineated the molecular details of the Bd0108:Bd0109 interaction, showing both the biochemical mechanism and many of the residues of Bd0108 that facilitate the interaction with Bd0109. Binding experiments with ITC (Fig. **7**) demonstrate a low affinity interaction with a stoichiometry of 3, suggesting that Bd0109 may be able to accommodate the binding of multiple Bd0108 proteins, perhaps without strict preference for an individual conformation. This is typical of IDPs, which can bind their targets in multiple conformations with weak affinity [61] and/or with suboptimal energy [62]. We do not expect that the stoichiometry of 3∶1 comes from oligomeric Bd0108 (as observed in our gel filtration studies when the protein was presumably inadvertently oxidized). Furthermore, the ITC data show both favorable enthalpy and entropic contributions to the binding mechanism. This indicates that hydrogen bonding and likely electrostatics contribute, and that the hydrophobic residues of Bd0108 interact with Bd0109. The latter conclusion is supported by the NMR titration data (Fig. **8**), which shows that the N-terminal region of Bd0108 is a major site of interaction with Bd0109. The N-terminal region of Bd0108 (24–66) contains 8 out of the 10 hydrophobic residues in the Bd0108 sequence (residues 24–101) and displays many conserved charged and polar residues (Fig. **8D**). From this data we conclude that Bd0108 interacts with Bd0109 with low affinity via its N-terminal residues.

It is of further interest to note that during the NMR titration experiments the N-terminal region of Bd0108 entered into intermediate exchange, indicative of the different states of this process interconverting on the µsec to msec timescale [32]. This shows that unlike many IDPs, Bd0108 does not undergo a disorder to ordered transition upon binding Bd0109 [53]. Considering both the NMR data and the weak affinity of the complex revealed by ITC, the Bd0108:Bd0109 interaction may best be described as the ‘random model’ in the ‘fuzzy complex’ view of dynamic protein-protein interactions [63]. Fuzziness in protein-interactions refers to the observed spectrum of order to disorder observed in studied examples of protein-protein binding. The random model describes the far extreme of transient protein-protein interactions of low affinity in which one partner remains disordered in the bound state. The random model binding mode is most often observed in signaling interactions, such as T-cell signaling [63], [64]. Given this observation, it suggests that Bd0108 has evolved as an IDP to function in signaling or to facilitate multi-specificity for additional binding partners [48]. The multi-specificity hypothesis is supported by the fact that when Bd0108 and Bd0109 are co-expressed in *E. coli* they localize to the periplasm at the pole regions [14]. The leading pole is important in predation, as it senses prey and organizes elements of the invasion machinery [65]. Given the observed targeting, Bd0108 may be delivered by Bd0109 for interaction with a currently unknown binding partner.

HI strains reporting nonsense or frameshift mutations in *bd0108* [3], [14] indicate that these alterations occur throughout the entirety of the gene and hence Bd0108 function can be compromised in a variety of ways. Our study centers on the HD100 strain of *Bdellovibrio* and it is of note that the related strain *B. bacteriovorus* Tiberius is able to grow in HI and HD fashion at the same time, speculatively to take advantage of its highly-polluted environmental niche [66]. Our data and a sequence alignment of Bd0108 with the Tiberius equivalent Bdt_0101 indicate that this dual HI/HD phenomenon is unlikely to be attributed to alterations in this gene (Fig. **8D**).

What implications do our results have for other predators related to the classical HD100 strain? It is interesting that in the epibiotic predator *B. exovorus* JSS, which attaches to and consumes but does not enter prey, there are homologues to both Bd0108 and Bd0109. These gene products include A11Q_2207 and A11Q_2375, which have 38% and 62% sequence identity to Bd0109, and A11Q_2208, which has 28% sequence identity to Bd0108 [67]. Although A11Q_2375 has a higher agreement with Bd0109 and is in a locus with Tad genes, we suggest that the synteny of A11Q_2207/2208 provide the stronger equivalent of the hit locus rather than the distal A11Q_2375 gene product. However, this does not preclude that *B. exovorus* may have two functional Bd0109 homologues which perhaps play a role in the different prey phenotype observed relative to *B. bacteriovorus.* In this regard, it is interesting that it has proven difficult/impossible to obtain HI variants of this bacterium [67]. Moreover, the alignment of A11Q_2208 with Bd0108 indicates two immediately apparent gross features (Fig. **8D**). A11Q_2208 conserves the CXXC motif and surrounding residues, but importantly lacks the conserved N-terminal region of Bd0108 that we show participates in the interaction with Bd0109. Conversely, *Bdellovibrio* strain W, which phenotypically is able to form sporulation state-like stalled bdelloplasts [68], possesses a Bd0108 homologue lacking the C-terminal region of the protein. The scenario in the marine *Bdellovibrio*-like organism *Bacteriovorax marinus* SJ is also complex. The *B. marinus* SJ Bd0109 homologue BMS_0182 (35% sequence identity) has two small neighbors with putative signal peptides (BMS_0183/4) that we find have no significant homology to Bd0108 [69]. HI-like growth has been reported for strain SJ [70], which we postulate will arise from a different mutational mechanism akin to *B. bacteriovorus* HI variants with a wild type *bd0108* sequence.

We have also used the recently-solved structures of RHS proteins to gain a better idea of the distribution of repeats in Bd0109 – the hidden Markov model-based server HHPred [71] suggests that residues 125 onward of Bd0109 contain continuous RHS repeats (Fig. **1**). Recent structural work with an ABC toxin from *Yersinia entomophaga* has shown that RHS repeat domain proteins utilize the YD repeat as a structural motif to form a large hollow particle (59,000 Å^3^) built from a continuous β-sheet [24]. Effectively this creates a large shell with the proposed function of encapsulation of proteins, both for the protection of the cargo from proteases and as part of the secretion mechanism. The modularity of typical RHS proteins positions the repeats at the N-terminus of the protein, followed by a protease domain, and then a variable region (that is often toxic in nature). The current working model suggests that the repeats chaperone the toxin, respond to a stimulus, activate the protease functionality and then “deliver” the toxin from the end of the barrel [24]. In this regard, it is intriguing that Bd0109 appears to possess no protease or C-terminal variable domain; this situation has some precedent in other organisms where RHS genes recombine to “swap” tips [72]. To the best of our knowledge, we cannot find an RHS family member with assigned function in the literature that is lacking the protease and C-terminal variable regions. Structural determination of Bd0109 will clear up this mystery, and also possibly reveal the role of the N-terminal domain, which at present cannot be predicted with confidence. It is also of interest that the closely-related dual HD/HI strain Tiberius has two extra RHS genes in comparison to HD100, one of which akin to Bd0109 lacks the C-terminal protease/toxin domains. Given the continuity of RHS repeats in Bd0109 and the random model protein-protein interaction we observe between Bd0108 and Bd0109, we hypothesize that Bd0109 encapsulates Bd0108. Additionally, as it is known that IDPs undergo compression in crowded environments [73]; this model is compatible with our ITC data and NMR titration data – the mixed hydrophobic and hydrogen-bonding interactions we observe would be typical of the inward-facing residues of the RHS repeats. The role of this structural complex requires further study, but we can speculate that this system might act conventionally (Bd0109 delivering Bd0108, possibly to pili) or in reverse (Bd0109 “receives” Bd0108 as part of a signaling response; it has been shown that there is a quorum-sensing like character to HI growth [9]). This latter possibility would presumably be analogous to the signaling required in ABC toxins for protein release from the RHS component [24]; in this regard Bd0109 would have an as yet unidentified payload. The (extra)cellular localization of Bd0108 has yet to be determined unequivocally, and there remains the possibility that it could undergo a shift from IDP to folded unit upon successful transit; we note that there appear to be no consensus sequence motifs among Bd0108 homologs for post-translational modification.

Here we have revealed that the predatory lifecycle of *Bdellovibrio bacteriovorus* is governed by an intrinsically disordered protein. Additionally, we propose that this mechanism involves the encapsulation of an IDP by an RHS family domain, analogous to RHS usage in non-predatory bacteria.

## Materials and Methods

### Molecular cloning of Bd0108 and Bd0109 expression constructs

The pET26b pelB periplasmic expression strains of Bd0108 and Bd0109 were identical to those documented previously [14]. Cytoplasmically-expressed N-terminal thrombin cleavable histidine-tagged Bd0108 was made via a similar restriction-free protocol, utilizing the primer pair 5′-gcagcggcctggtgccgcgcggcagc catatggctgacgaaaatgccaaccgcccg-3′ and 5′-ctcagtggtggtggtggtggtgctc gagttactgtcttccagtcccggc-3′ to clone amino acids 23–101 into pET28b (this added the tripeptide SHM to mature N-terminus after thrombinization).

### Purification of Bd0108 and Bd0109

The expression constructs were transformed into Bl21(DE3) cells, grown at 37°C in either M9 minimal or LB media. Protein expression was induced at A_600_>0.6 with the addition of 1 mM IPTG (isopropyl 1-thio-β-D-galactopryanoside) and were further allowed to grow for 20 h at 20°C. Isotopic labeling of Bd0108 was performed with M9 minimal media substituted with 1 g/L ^15^NH_4_Cl and/or 3 g/L (^13^C_6_)glucose. After expression, cells were harvested, lysed by an Emulsiflex C5 (Avestin), and the lysate cleared by centrifugation at 16000 rpm for 30 minutes. The lysate was then passed over NTA-sepharose beads (Pierce) at 25°C, washed with 50 column volumes of 50 mM Tris(2-carboxyethyl)phosphine pH 7.5 500 mM NaCl 25 mM Imidazole, and protein eluted in the same buffer with the imidazole concentration increased to 500 mM. After the elution of Bd0108, 1 mM of β-ME (β-mercaptoethanol) was added immediately. Bd0108 was digested O/N at 4°C with 1/50 ratio of Thrombin to remove the 6 His-tag and simultaneously dialyzed into 20 mM Tris pH 7.5 200 nM NaCl 1 mM β-ME. The reaction was concentrated and undigested material removed by a second pass over an NTA column. The resulting flow-through containing cleaved Bd0108 was concentrated and further purified by gel-filtration using an SD75 column (GE Healthcare) equilibrated in 20 mM Tris pH 7.0 100 mM NaCl 2 mM β-ME. The estimated molecular weight of Bd0108 was compared to a standard curve generated from protein standards (GE healthcare) run in the Bd0108 gel filtration buffer. Bd0109 was purified as described in the previously established protocol [14] then dialyzed O/N at 4°C into 20 mM Tris pH 7.0 100 mM NaCl 2 mM β-ME. All buffers used were chilled to 4°C.

### Dynamic Light Scattering

Purified Bd0108 was injected into a DynaPro-801 (Protein Solutions) using a syringe with a 0.22 µm filter at 2 mg/mL in 20 mM Tris pH 7.0 100 mM NaCl 2 mM β-ME. The protein sample was also centrifuged at 6000 rpm for 15 minutes before injection to remove any larger precipitates. Data was analyzed with the provided software as mono-modal using an aqueous buffer model. The molecular weight was calculated from the internal standard curve of proteins.

### Disulfide Aggregation Assay

Bd0108 before purification by gel-filtration and omitting the initial addition of reducing agent was incubated with and without 1 M β-ME for 10 minutes at 25°C. After incubation, protein-loading dye (Biorad) was added to each sample, with and without reducing agent. The designated samples were boiled for 5 minutes at 95°C and all samples centrifuged for 5 minutes at 16000 rpm before loading onto a 17% SDS-PAGE gel for separation. After separation the gel was visualized by staining with Coomassie-dye.

### NMR Spectroscopy

NMR spectra for backbone assignment were recorded at 25°C on a Bruker 600 MHz DRX spectrometer equipped with a 5 mm inverse TXI cryogenic probe. Relaxation and NOE NMR experiments were recorded on a Bruker 800 MHz AVANCE spectrometer, and titration NMR experiments recorded on a Bruker 900 MHz AVANCE spectrometer equipped with a 5 mm inverse TCI cryogenic probe. The Bd0108 sample was 1 mM Bd0108 in 20 mM Tris pH 7.0 100 mM NaCl 2 mM β-ME supplemented with 5% D_2_O. The ^1^H, ^13^C, ^15^N nuclei of isotopically labeled Bd0108 were detected and assigned by standard heteronuclear NMR experiments [74]. Backbone resonances were assigned using hncacb, cbcaconnh, hnco, hncaco, and hnha spectra. Aliphatic side chains were assigned using a ccconh experiment, and hydrogens assigned from ^15^N-TOCSY-HSQC and the ^15^N-NOESY-HSQC spectra. Backbone dihedral angles and secondary structure were predicted using the assigned chemical shift data with Talos+ [34]. A three-dimensional ^15^N-NOESY-HSQC was collected with a 100 ms mixing time on a [35]. Relaxation data for Bd0108 were collected at 25°C following established methods using a 5 second recycle delay for the ^1^H-^15^N NOE relaxation experiment and a 2 second recycle delay for T_1_ and T_2_ measurements [43]. NMR titration experiments were performed with purified Bd0108 and Bd0109 after dialysis into the same buffer stock (20 mM Tris pH 7.0 100 mM NaCl 1 mM β-ME). ^1^H-^15^N-HSQC were recorded for 25 µM Bd0108 in the presence of 0, 25, 100, and 200 µM Bd0109. All NMR data was processed using NMRPipe [75] and analyzed with Sparky (T.D. Goddard and D.G. Kneller, Sparky 3 University of San Francisco).

### Circular Dichroism Spectroscopy

Purified Bd0108 was diluted to 15 µg/mL in 20 mM KHPO_4_ at pH 6.5, pH 7.0, and pH 7.5 and placed in a 0.2 cm path length cuvette. Spectra were collected on a Jasco-715 or Jasco-815 from a wavelength of 190 nm to 260 nm. Background spectra consisting of Bd0108 buffer diluted into 20 mM KHPO_4_ was subtracted from the data. Data were analyzed using Dichroweb [38] with a mean residue weight of 104 Daltons for deconvolution of secondary structure partitions and plotted as mean residue ellipticity.

### Isothermal Titration Calorimetry

Purified Bd0108 and Bd0109 were dialyzed overnight at 4°C into the same buffer stock of 20 mM Tris pH 7.0 100 mM NaCl 1 mM β-ME. Bd0108 was concentrated to 1.2 mM and Bd0109 used at 0.02 mM. All experiments were performed using a VP-ITC calorimeter (GE Healthcare) at 25°C. The Bd0108 control was Bd0108 titrated into buffer alone, whereas the Bd0109 control was buffer titrated into Bd0109. The final heats of binding were analyzed using Origin Software (GE Healthcare) using a one-site model.

## Supporting Information

S1 Figure **Metal Binding Prediction.** The amino acid sequence of the Bd0108 cytoplasmic construct not including cloning artifacts was submitted to the metaldetector server (http://metaldetector.dsi.unifi.it/). The resulting bioinformatics analysis predicts that the two cysteine residues of Bd0108 form a disulfide.
(TIF)

S2 Figure **Control ^1^H-^15^N HSQC spectra.** (A) Overlay of Bd0108 taken at 0 days pH 6.0 and pH 7.0 and 2 months at pH 7.0. (B) Overlay of spectra obtained at 25°C and 40°C from the PelB construct. (C) Overlay of spectra obtained from Bd0108 produced in the cytosol (blue) or tagged with PelB for delivery into the periplasm (red). The extra resonances observed in the PelB construct are due to the additional cloning artifacts relative to the cytoplasmic construct.
(TIF)

S3 Figure **CS-Rosetta Calculation.** Shown is the resulting output of the clustering of similar energy structures. The calculation fails to simulate the folding of a Bd0108 structure or structures with a low rmsd and similar energy minima.
(TIF)
