# Supplementary figures and images for: The Lifestyle Switch Protein Bd0108 of Bdellovibrio bacteriovorus Is an Intrinsically Disordered Protein
Source: PLoS One. 2014 Dec 16;9(12):e115390. doi: 10.1371/journal.pone.0115390 (PMC4267844; doi:10.1371/journal.pone.0115390)

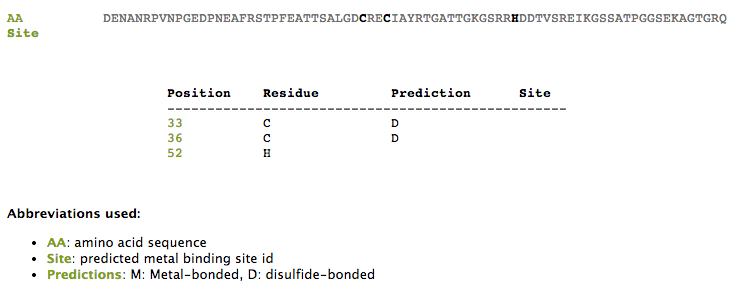

Supplement: S1 Figure — Metal Binding Prediction. The amino acid sequence of the Bd0108 cytoplasmic construct not including cloning artifacts was submitted to the metaldetector server (http://metaldetector.dsi.unifi.it/). The resulting bioinformatics analysis predicts that the two cysteine residues of Bd0108 form a disulfide. (TIF) [file pone.0115390.s001.tif]

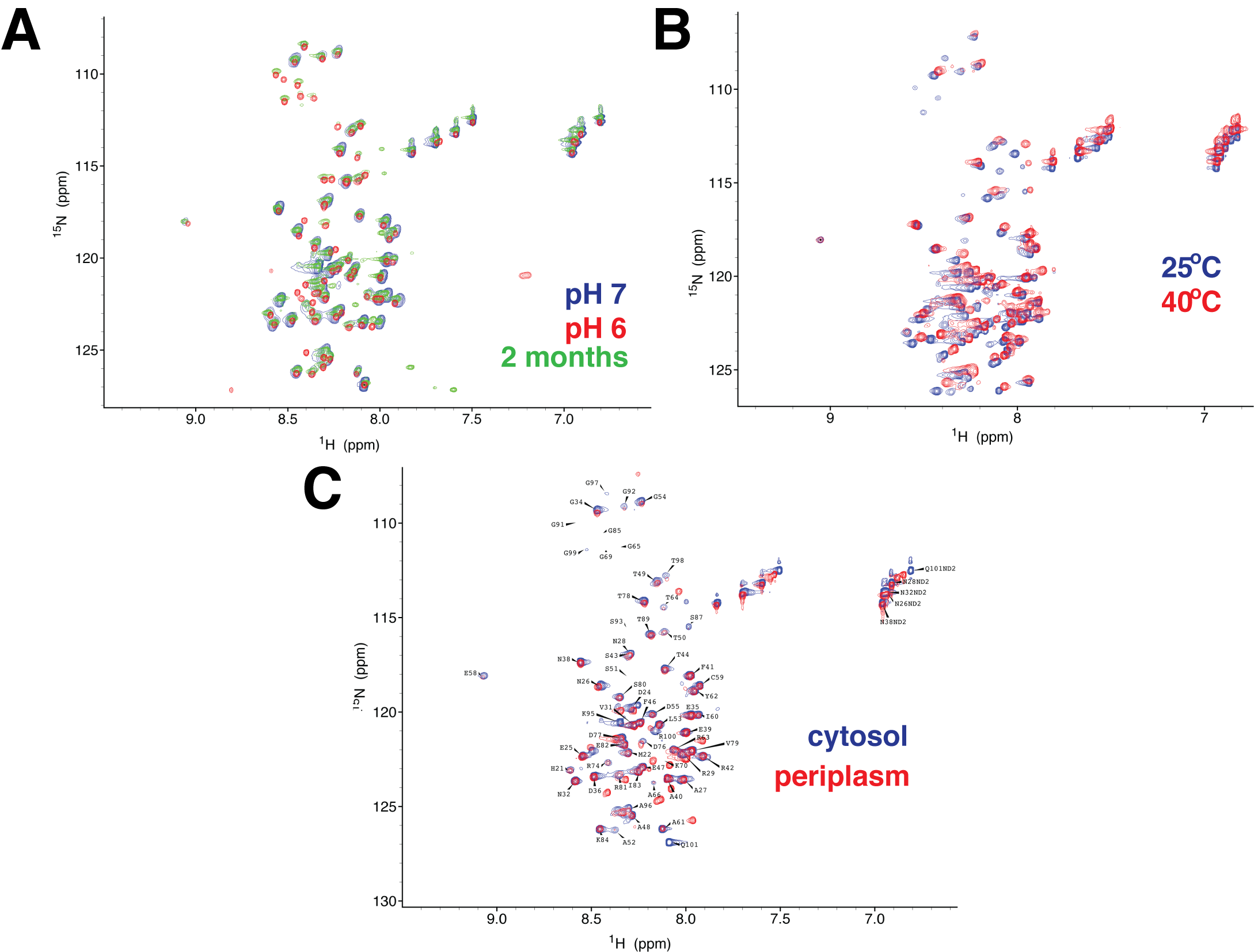

Supplement: S2 Figure — Control 1H-15N HSQC spectra. (A) Overlay of Bd0108 taken at 0 days pH 6.0 and pH 7.0 and 2 months at pH 7.0. (B) Overlay of spectra obtained at 25°C and 40°C from the PelB construct. (C) Overlay of spectra obtained from Bd0108 produced in the cytosol (blue) or tagged with PelB for delivery into the periplasm (red). The extra resonances observed in the PelB construct are due to the additional cloning artifacts relative to the cytoplasmic construct. (TIF) [file pone.0115390.s002.tif]
